# Twelve-Month Follow-Up After the Treatment of Periodontal Conditions Using Scaling and Root Planning Alone vs. Laser-Assisted New Attachment Procedure

**DOI:** 10.3390/diagnostics15141799

**Published:** 2025-07-16

**Authors:** Edwin Sever Bechir, Farah Bechir, Mircea Suciu, Anamaria Bechir, Andrada Camelia Nicolau

**Affiliations:** 1 Faculty of Dental Medicine, George Emil Palade University of Medicine, Pharmacy, Science, and Technology of Târgu Mureș, 38 Gh. Marinescu Str., 540142 Târgu Mureș, Romania; edwin.bechir@umfst.ro (E.S.B.); mircea.suciu@umfst.ro (M.S.); 2 Faculty of Dental Medicine, “Titu Maiorescu” University of Bucharest, 67A Gh. Petrascu Street, 031592 Bucharest, Romania; anamaria.bechir@gmail.com; 3 Faculty of Medicine, Transilvania University of Brașov, 56 Nicolae Bălcescu Str., 500019 Brașov, Romania; andrada.nicolau@yahoo.com

**Keywords:** periodontal disease, scaling and root planing (SRP), laser-assisted new attachment procedure (LANAP), Nd:YAG laser treatment

## Abstract

**Background/Objectives**: Periodontitis is a chronic inflammation of the periodontium that induces damage in the periodontal ligaments and the surrounding alveolar bone. This study aimed to comparatively evaluate the clinical outcomes of two therapies used in the management of periodontal conditions, represented by scaling and root planing (SRP) alone and laser-assisted new attachment procedure (LANAP). **Methods:** Two quadrants of the oral cavity from each selected patient were randomly allocated to one of the treatment groups, SRP or LANAP. The periodontal status was documented in a periodontal chart at baseline, six weeks, and one year after treatment. SRP was performed in the first group of patients. The LANAP protocol was carried out on the patients belonging to the second group. **Results:** The outcomes of the study highlighted that LANAP leads to a reduction in periodontal disease signs (pocket depth, bleeding on probing, and gingival recession), contributing to the formation of new attachment tissues. LANAP shows more stability in maintaining the improvements achieved during six weeks, while SRP shows a slight deterioration in several parameters, particularly attachment loss, between six weeks and one year. The collected data at six-week and one-year follow-ups show improvements in periodontal health, thus improving oral health. **Conclusions:** Both minimally invasive periodontal procedures were effective, with LANAP demonstrating greater efficiency in patients with chronic periodontal disease, a greater reduction in pocket depth, and improved clinical outcomes compared to SRP alone.

## 1. Introduction

Periodontitis, a chronic inflammation of the periodontium, induces damage in the periodontal ligaments and the surrounding alveolar bone [1]. The severity and extent of periodontal disease are influenced by genetic and environmental factors [2,3]. This condition affects more than 40% of the world population [3,4,5]. It is characterized by continuous damage to the periodontal soft and hard tissues and represents an essential concern for global public health [6,7] that can induce systemic conditions [8].

The predominance of periodontitis increases dramatically in adults aged between 50 and 60 years old, and it is expected to grow due to the globally aging population [9].

This condition impacts individuals prone to extensive tooth loss, thus inducing physiognomic and masticatory dysfunctions. Collectively, they negatively impact the patients’ nutrition, quality of life, and self-esteem, with significant socio-economic consequences [10,11,12].

The actual classification of periodontal and peri-implant diseases has considerable consequences for dental practice. This classification is made by the staging and grading of the gum condition. The stages (severity) and the extension (grade) of disease are classified based on the measurable degree of destruction in the damaged tissue. The development of periodontitis is categorized into stages I to IV (where stage I is the mildest, stage IV is the most severe), while the disease complexity is classified into grades A, B, or C (where grade A is the lowest risk, and grade C is the highest risk of progression rate) [12,13,14].

In periodontitis, there is a coexistence of an imbalance of the natural oral microbiota and the dysbiosis (reduced resistance) of the host, with inflammatory changes in the surrounding alveolar bone and of the connective tissue [15]. This is one of the most frequent chronic inflammatory and/or infectious oral conditions caused by bacteria [16,17]. The microbial dysbiosis in the biofilm of dental plaque represents the main cause of the periodontal disease onset [6]. The progression of this condition is induced by the host’s specific response [18].

Clinical attachment loss (CAL) represents an indicator of destructive periodontal disease and emerges in sites where clinical signs of gingival inflammation and connective tissue attachment loss are present [19]. In advanced periodontal disease, due to the decrease in the periodontium clinical attachment level, the pathological mobility of the affected teeth occurs first, followed by their loss [1].

Decreasing the inflammatory and infectious processes and also ceasing the periodontal tissue destruction represent the main goals for the treatment of periodontal conditions [5]. The most commonly accepted clinical approach in treating periodontal disease remains the conventional, non-surgical treatment, which consists of the elimination of the dental biofilm and calculus by scaling and root planing (SRP) [6,18], followed by long-term monitoring aiming to maintain the obtained results [20].

SRP is a deep dental debridement technique, consisting of removing the calculus and bacteria that build up around the tooth roots under the gum line with an ultrasonic scaler and then manually with a metal curette. SRP aims to suspend the spreading of infection, reduce the periodontal inflammation, and allow the reformation of periodontal attachment in favor of pocket healing [21].

The laser-assisted new attachment procedure (LANAP) is a minimally invasive procedure that disintegrates the bacteria inside the periodontal pockets and eliminates the compromised periodontal tissues by disinfecting the affected areas, therefore promoting periodontal tissue regeneration and attachment of newly formed connective tissues to the cleaned root surfaces. However, the major disadvantage of the Nd:YAG laser beam is that it cannot detach the dental calculus located in difficult-to-reach areas [5,22,23].

Numerous studies have underlined the favorable results of SRP combined with adjunctive therapies such as lasers, antibiotics, local drug delivery, or ozone. In the systematic review conducted by Mahdizade Ari et al. [24], clinical trials in treating chronic periodontal conditions published between the years 2008 and 2023 were evaluated in order to establish the efficacy of photodynamic therapy as an adjunctive treatment to the SRP alone. The authors concluded that photodynamic periodontal therapy is an encouraging complementary therapeutic method effective in reducing chronic periodontal conditions, with low costs and well-supported by patients. Pardo et al. [25] investigated the outcomes of using photodynamic treatments associated with nonsurgical periodontal therapy and observed an improvement in the studied periodontal index after three months. Other studies suggest that associating SRP with the ozone therapy may lead to beneficial outcomes in decreasing inflammation and the depth of periodontal pockets in comparison with SRP as monotherapy. Scribante et al. [26] assessed the efficiency of SRP associated with ozonized gels in periodontal conditions, compared to conventional chlorhexidine for at-home use. The authors proposed ozone as an alternative to chlorhexidine.

The aim of the study was to evaluate and compare the clinical outcomes of scaling and root planing as a monotherapy versus the laser-assisted new attachment procedure, at six weeks and one year following the completion of the therapies, in teeth presenting at least 4 mm depth periodontal pockets on at least one of the six inspected dental surfaces.

## 2. Materials and Methods

### 2.1. Ethical Approval

The study was carried out in conformity with the ethical principles of the Declaration of Helsinki and good clinical practice. The study protocol was approved by the Ethics Committee of “George Emil Palade” University of Medicine, Pharmacy, Science, and Technology of Târgu Mureș, Romania (number 750 from 18 February 2020). The study was performed in the Integrated Dental Center of the Dental Medicine Faculty of the University and Dentaltop Dentistry Clinic in Târgu Mureș between February 2020 and October 2024, with an intermission demanded by the circumstances of the COVID-19 pandemic.

### 2.2. Participants

The selected patients were informed about the requisites of the study, and solely those who voluntarily accepted the demands of the program were accepted. The phases of the study and the need for assessment were explained to every participant. Before the start of the study, written informed consent was obtained from all participants regarding the use of their data and samples for scientific purposes.

In all eligible selected patients, rigorous and accurate anamneses (name, age, address, job, allergies, dietary manners, smoking, parafunctional/vicious habits, and acute and/or chronic illnesses) were recorded, as well as clinical examinations, followed by X-ray examinations (OPG).

### 2.3. Inclusion and Exclusion Criteria

The oral examinations were performed by a single examiner in order to avert the calibration mistakes and included the inspection of the oral tissues, the evaluation of oral hygiene, the location, and degree of periodontal disease.

For all selected teeth, periodontal charting including probing depths, clinical attachment level (CAL), visible plaque index, bleeding on the probing, and tooth mobility degree was performed at six sites per tooth. Also, the orthopantomograms of all selected patients were examined and noted in the file.

The inclusion and exclusion criteria of the study are presented in Table 1.

### 2.4. Clinical Procedure

All patients were accepted into the study in accordance with the same criteria. They presented at least two teeth with more than 4 mm deep periodontal pockets, located in distinct quadrants. Of the 15 selected patients (30 teeth with deep periodontal pockets), one patient withdrew. Of the remaining 14 participants (28 teeth), 7 were females and 7 were males, aged between 36 and 67 years old, with a median age of 51.5 ± 15.5 years.

The clinical protocol included a specialty consultation to evaluate patient eligibility; periodontal parameters recording in the periodontal chart and OPGs. The patients were informed about the stage of their condition. The selection of patients was conducted in accordance with the inclusion and exclusion criteria followed by presentation of the study requirements and of the treatment plan. After obtaining informed consent, professional dental cleaning was performed. Subsequently, the teeth of the selected patients were randomly allocated to one of the two study groups (SRP and LANAP).

All patients were trained on the correct methods regarding at-home dental hygiene. They received all the post-interventional instructions and all the indications regarding tooth brushing (with soft toothbrushes and using fluoride toothpaste) and chlorhexidine mouthwash rinses (twice a day, for two weeks). The observed clinical parameters were registered in the periodontal chart at baseline, six weeks after treatment, and one year after treatment.

An ultrasonic scaler was used for calculus removal: the Satelec Newtron (Acteon^®^ Group, Jersey City, NJ, USA). This procedure was completed using rotary dental brushes, prophylactic paste, and airflow (Air-N-Go Airflow—Acteon^®^ Group, Jersey).

SRP was performed in the first group (7 patients, 14 teeth), as well as on the two remaining quadrants that were not included in the study, under local anesthesia. The procedure consisted of manual instrumentation using area-specific Gracey curettes (HuFriedy^®^ Group, Chicago, IL, USA). The procedure was continued until a smooth root surface was achieved, free of visible or tactile signs of calculus or altered root cementum.

The LANAP protocol was performed using an Nd:YAG laser with a wavelength of 1064 nm, type Lightwalker AT-S, Fotona^®^, Ljubljana, Slovenia, a specialized comprehensive dental laser. For Nd:YAG, the wavelength is 1064 nm. The longer pulses (600–1000 µs) are used for soft tissue. The utilized applicators for dental use can be represented by R23-A Nd:YAG (1064 nm) or R24-C Nd:YAG (1064 nm) [27].

The first application of the Nd:YAG laser beam was conducted under local anesthesia, using a power setting of 2.5 W with an MSP pulse (100 μs duration and 20 Hz frequency) for 20 s. The 0.3 mm diameter optical fiber was inserted into the gingival sulcus parallel to the long axis of the tooth, and positioned 1 mm short of the base of the periodontal pocket, in order to ablate the altered sulcular epithelium. Lateral and apical sweeping movements were performed, followed by conventional scaling and root planning. Subsequently, the Nd:YAG laser was applied for the second time, with a power of 3.5 W and a VLP pulse of 600 μs duration and 20 Hz frequency, for 20 s per tooth, aiming to induce coagulation and promote the formation of a stable clot to seal the periodontal pocket.

The patient training program focused on educating and motivating individuals to effectively perform oral hygiene practices twice daily, at home. Alongside the oral hygiene instructions, patients were advised to use a 0.12% chlorhexidine solution (Curasept ADS^®^, Curaden AG, Kriens, Switzerland) twice a day for a duration of two weeks following the interventions to support plaque control.

Clinical outcomes of scaling and root planing as monotherapy and LANAP were performed at baseline (upon patients’ presentation), six weeks post-treatment, and one year post-treatment.

**Figure 1:**
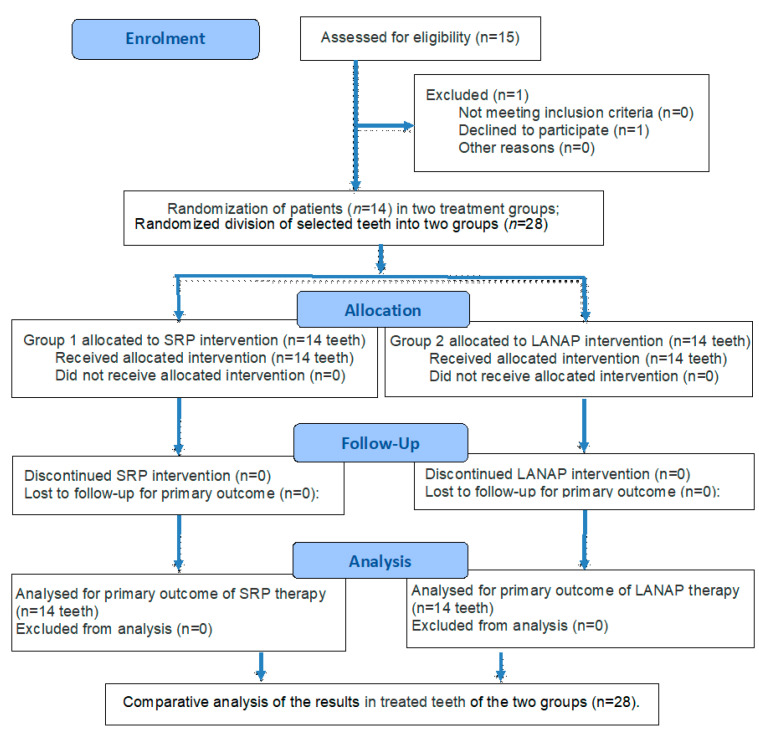
The flow diagram of the study.

The flow diagram of the study based on CONSORT 2025 [28] is presented in Figure 1.

### 2.5. Statistical Analysis

The statistical analysis was performed in the Microsoft Excel 365 program. Two-tailed, paired sample t-tests were performed to compare the post-treatment outcomes at six weeks and one year, as well as the change in any of the clinical markers between six weeks and one year post-treatment, for the comparison of the treatment efficiency. The significance level was considered 0.05.

## 3. Results

By comparing the progression of the periodontal parameters at six weeks and at one year following the treatment with either SRP alone or LANAP, a comparative evaluation of their impacts on periodontal health over time was carried out.

The evolution of clinical periodontal parameters with *p* values after six weeks post-treatment is detailed in Table 2, and after one year post-treatment in Table 3.

Comparing the progression of the clinical parameters between six weeks and one year post-treatment for SRP and LANAP, the following observations can be made:

- % plaque index:
  - SRP: The plaque percentage increases significantly from 1.00% at six weeks to 3.71% at one year (*p* = 0.026). This shows a noticeable increase in plaque accumulation over the 12 months, though it does not return to the original levels before treatment.
  - LANAP: The plaque index increases from 0.93% at six weeks to 1.71% at one year. However, this change is not statistically significant (*p* = 0.102), indicating that there is a slight rise in plaque accumulation over the year, but not a substantial one.
- % bleeding on probing:
  - SRP: The percentage of bleeding on probing increases from 1.29% at six weeks to 2.71% at one year (*p* = 0.05), which is statistically significant. This suggests that the bleeding on probing is still slightly higher at one year, though it is still much lower than before treatment.
  - LANAP: The percentage of bleeding on probing increases from 1.29% at six weeks to 1.86% at one year. This change is not statistically significant (*p* = 0.071), indicating a small rise in bleeding on probing, but it remains relatively low compared to baseline.
- Periodontal probing depth (PPD):
  - SRP: The score increases from 0.46 at six weeks to 0.54 at one year (*p* = 0.006), which is statistically significant.
  - LANAP: The score increases from 0.49 at six weeks to 0.52 at one year. This change is not significant (*p* = 0.165).
- Attachment Loss:
  - SRP: Attachment loss worsens slightly from −0.54 mm at six weeks to −0.64 mm at one year (*p* = 0.016). This change is statistically significant, indicating a slight loss of attachment over the 12-month period after SRP treatment.
  - LANAP: Attachment loss remains relatively stable, with a minor improvement from −0.52 mm at six weeks to −0.56 mm at one year. The change is not significant (*p* = 0.054), indicating that the positive effects on attachment loss from LANAP are maintained over the year.

In conclusion, it was observed that there is a slight increase in plaque and bleeding between six weeks and one year, but the results remain relatively stable, with no significant changes in attachment loss for LANAP. The periodontal probing depth (PPD) remains consistent.

There is a more significant increase in plaque and bleeding from six weeks to one year for SRP. Additionally, there is a slight loss of attachment at one year compared to six weeks.

Thus, LANAP shows more stability in maintaining the improvements achieved at six weeks, while SRP shows a slight deterioration in several parameters, particularly attachment loss, between six weeks and one year.

**Figure 2:**
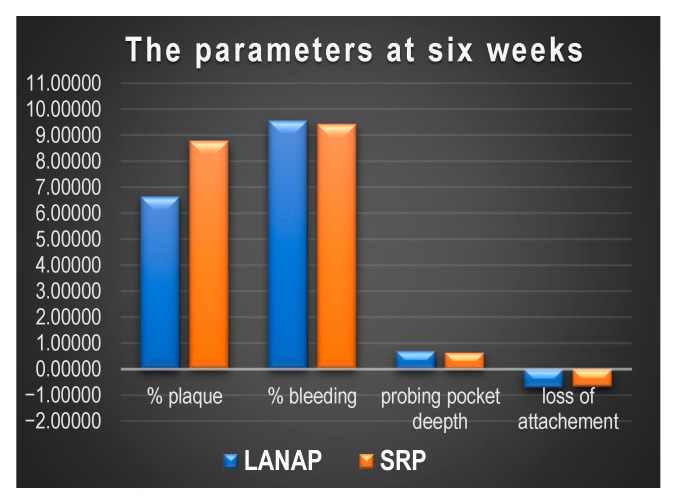
The periodontal parameters after six weeks post-treatment.

**Figure 3:**
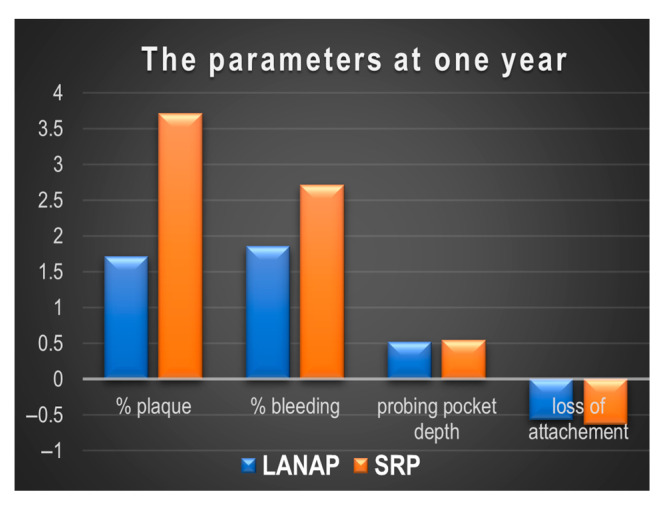
The periodontal parameters after one year post-treatment.

The evolution of the parameters at six weeks and one year post-treatment is shown in Figure 2 and Figure 3.

Comparing the progression of the clinical parameters from immediately post-treatment to one year following treatment for SRP and LANAP, the following observations were noted (Table 4):

- % plaque index:
  - SRP: The plaque percentage significantly decreases at six weeks (from 8.79% to 1.00%) but increases significantly at one year (3.71%) compared to six weeks (*p* = 0.026). This suggests a return of plaque to a higher level at one year, though it does not reach the initial values.
  - LANAP: Similarly, after a significant reduction in plaque at six weeks (from 6.64% to 0.93%), the plaque percentage slightly increases at one year (1.71%), but this increase is not statistically significant (*p* = 0.102).
- % bleeding on probing:
  - SRP: Bleeding decreases significantly at six weeks (from 9.43% to 1.29%) and increases slightly at one year (2.71%), with a significant difference (*p* = 0.0498), indicating a return to a higher bleeding level compared to six weeks.
  - LANAP: Similarly, bleeding on probing significantly decreases after treatment at six weeks (from 9.57% to 1.29%) and remains slightly elevated at one year (1.86%), but the difference is not statistically significant (*p* = 0.071).
- Periodontal pocket depth (PPD):
  - SRP: PPD decreases significantly at six weeks (from 0.63 to 0.46), but increases at one year (0.54), with a significant change compared to six weeks (*p* = 0.006), suggesting an increase in medication use at one year.
  - LANAP: PPD significantly decreases at six weeks (from 0.69 to 0.49) but remains almost constant at one year (0.52), with no significant change between six weeks and one year (*p* = 0.165).
- Attachment Loss:
  - SRP: Attachment loss improves significantly at six weeks (from −0.67 mm to −0.54 mm) but improves less at one year (from −0.54 mm to −0.64 mm), with a significant change between six weeks and one year (*p* = 0.016), suggesting a slight loss of attachment at one year compared to six weeks.
  - LANAP: Attachment loss improves significantly at six weeks (from −0.72 mm to −0.52 mm), and this improvement remains almost constant at one year (from −0.52 mm to −0.56 mm), with no significant change (*p* = 0.054).

In conclusion, LANAP shows a significant reduction in plaque and bleeding at six weeks, with these improvements being maintained at one year, though there is a slight return of plaque and bleeding. SRP also shows significant reductions in plaque and bleeding at six weeks, but these effects are not as durable over the long term, with plaque and bleeding increasing at one year. Regarding attachment loss, both treatments showed positive results at six weeks, with improvements remaining constant at one year for LANAP, but slight attachment loss at one year for SRP.

The comparative outcomes of the study indicated that LANAP therapy reduces the signs of periodontal disease (pocket depth, bleeding on probing, and gingival recession), promoting the formation of new attachment tissues. Both treatments yielded significant improvements in the short term (six weeks), although LANAP appeared to be more effective in sustaining long-term results.

All these outcomes reflect the short-term and long-term efficacy of the two treatments at the specified follow-up intervals (six and twelve months). The obtained scores at six weeks and one-year follow-up induced an overall enhancement of patients’ periodontal health, thus improving their oral health status.

## 4. Discussion

The immediate aim of periodontal condition treatment is to impede, to confine, to control, or to remove the disease. The ideal purpose is to promote periodontal tissue healing.

The severity and extent of the periodontal conditions are correlated with the measurable degree of damaged tissues [24]. By their evolution, periodontal conditions are classified in stages I to IV, while by the disease complexity, they are subclassified in Grades A, B, or C [29,30,31].

The classification of periodontal conditions by staging and grading allows the correct periodontal diagnosis and offers the clinician the possibility of performing an individualized diagnosis and treatment plan for every patient [21,32]. The prediction of periodontal disease is related to the stage and grade of this condition, the existence of additional factors, and the applied treatment [6,33,34].

This study revealed that both therapies effectively reduce clinical signs of periodontal disease, including dental plaque, bleeding on probing, pocket depth, and attachment loss, thus improving patients’ oral and general health. Notably, LANAP therapy further contributed to the new attachment formation in the periodontal tissues. Currently, LANAP shows greater potential for long-term success compared to conventional periodontal approaches.

Many authors [35,36,37] studied the effects of diode laser therapy and scaling and root planing in chronic periodontitis. Subedi et al. [38] studied 30 patients affected by chronic periodontitis, with clinical attachment loss of more than 3 mm at one and three months posttreatment. The treatment showed significant improvements in clinical parameters at three months postoperatively (*p* ≤ 0.05), with improvement observed in the test site (*p* ≤ 0.05). Altalhi et al. [21] performed a comparative review regarding SRP and various types of laser therapy and underlined the bactericidal and decontamination effects of lasers compared to those of traditional SRP. They concluded that Nd:YAG lasers are not able to remove the totality of the dental calculus and can cause thermal damage if not used properly. They underlined that in order to prevent thermal damage, the used ideal power level for Nd:YAG laser therapy is 100 mJ/pulse. In our study, the applied Nd:YAG laser beams were used with a power setting of 2.5 W with an MSP pulse (100 μs duration and 20 Hz frequency) for 20 s. Yu et al. [39] found that gender and age influence the periodontal scores after full-mouth LANAP treatment. In the study performed by El Mobadder et al. [40], it is highlighted that the use of lasers presents several important advantages, such as an increase in the decontamination capacity of the periodontal pockets and surrounding tissues, dental biofilm, and the antibacterial effect concerning the periodontal pathogens. Yukna [41] evaluated LANAP as monotherapy in 22 patients affected by moderate to severe periodontitis, with probing depths (PD) up to 11 mm. The LANAP treatment used a 1064 nm Nd:YAG laser in a single-session active therapy. Following 12–18 months, the periodontal condition of the patients (PD, clinical attachment level, and furcation involvement) was reassessed and showed significant improvements. The author concluded that LANAP proved to be an effective and minimally invasive treatment for moderate to advanced periodontal diseases.

Periodontal diseases develop as a result of several factors, including both patient-specific risk factors and incorrect oral hygiene. The risk factors of periodontal disease can be subdivided into modifiable risk factors, including smoking tobacco, poor oral hygiene, diabetes mellitus, and pregnancy, and non-modifiable risk factors, like age and heredity, including genetic diseases [42,43]. Gender, age, missing teeth number, and tooth loss quantity during supportive periodontal therapy were linked to disease traits [29,44]. The higher the stage and the more rapidly progressive the grade, the worse the prognosis of the periodontal disease. Additionally, factors such as tobacco smoking and uncontrolled diabetes mellitus can affect the prognosis of the disease [33,44].

The results of non-surgical therapy show a significant reduction in tissue inflammation; however, several studies have demonstrated their limited efficacy as a sole method of treatment in periodontal pockets deeper than 5–7 mm, furcation areas, and generally hard-to-access locations [45]. In addition, the evaluation of laser as an adjunct to SRP is needed, as the two treatments are not mutually exclusive [46,47,48]. Acatrinei et al. [46] comparatively studied the effect of SRP with laser therapy and autologous plasma in the treatment of periodontal conditions, and they found that both options showed beneficial effects in decreasing the signs of periodontal conditions, with a low risk of relapse.

The use of laser beams in minimally invasive therapies of periodontal diseases is shown to be favorable. However, its application is indicated after a thorough conventional debridement, thus not being indicated as a substitution for SRP [40,49].

The majority of periodontitis types can be treated with nonsurgical or minimally invasive therapy, involving plaque control, scaling, and root planing (SRP); nevertheless, favorable results can be maintained with periodic recall and long-term monitoring [44,45,46,47,48,49,50].

The advantages of SRP in conjunction with adjuvant therapy, including lasers, antibiotics, local medication administration, or ozone, were highlighted in a number of investigations. According to Sareen et al. [51], ozone adjunct therapy with SRP may enhance all periodontal parameters than SRP alone, through reducing the periodontal probing (PI) and gingival index (GI). The clinical benefits of periodontal regenerative therapy have improved by the introduction of ozone therapy due to its antimicrobial and healing properties [52].

In order to reduce the long-term harm caused by incorrect periodontal therapy, it is advisable to take a proactive approach that combines regular follow-ups, probiotics, postbiotic ozonized chemicals, and traditional treatment of periodontal diseases. This synergistic effect has the potential to speed up recovery by reducing inflammation and restoring harmony to the oral microbiome [26,53,54,55,56,57,58,59].

Although periodontal diagnosis is often presented as a dimensional framework, it remains essential to establish an individualized diagnosis and treatment plan for every patient. Concurrently, it is necessary to apply the principles of P4 medicine (prediction, prevention, personalization, and participation for patient management) [60,61,62,63,64]. Moreover, the consequent periodontal maintenance therapy with long-term follow-ups is crucial in the success of periodontal disease treatment [64,65].

Further research will focus on the evaluation of the efficiency of SRP alone versus LANAP (Nd:YAG laser) and probiotics, postbiotic, and ozonized chemicals in the management of periodontal disease is warranted for the long-term follow-up of the obtained results.

## 5. Conclusions

Within the limitations of this study, it can be concluded that both minimally invasive periodontal procedures demonstrated clinical effectiveness; however, LANAP proved to be more efficient in patients with chronic periodontal disease, achieving a greater pocket depth reduction and improved clinical outcomes.

These findings suggest that LANAP holds greater potential for long-term success compared to SRP alone.

The clinical relevance of the findings is reflected in the therapeutic solutions available to dentists for managing teeth affected by periodontal diseases utilizing SRP and LANAP.

Our future goals in laser-assisted periodontal treatment include making it more personalized, precise, effective, and proactive, with faster healing.

## Figures and Tables

**Table 1:** Inclusion and exclusion criteria.

Inclusion Criteria	Exclusion Criteria
▪ Patients older than 18 years of age who agreed to partake in this study and signed the informed consent. ▪ Patients presenting a minimum of twelve natural teeth distributed in all four quadrants; ▪ Patients with a minimum of two teeth with periodontal pockets, in different quadrants, with at least 4 mm depth on at least one of the six inspected dental surfaces; ▪ Patients with stage II, grade B, localized periodontitis.	▪ Patients with periodontal treatment in the past twelve months; ▪ Patients with systemic and/or local therapy with antibiotics in the past six weeks; ▪ Patients with stage I or II, grade A, localized periodontitis; ▪ Systemic affections, which can modify the therapeutic result (diabetes, immune deficiencies, HBV, HCV, cancer, hematological disorders, epilepsy, etc.); ▪ Pregnancy and breastfeeding; ▪ Teeth with extraction indication; ▪ Incapacity or refusal to follow the study protocol.

**Table 2:** Evolution of the periodontal parameters with *p* values after six weeks post-treatment.

Variable	SRP	*p* Values SRP	LANAP	*p* Values LANAP
% plaque	1.00	0.0003	0.929	0.0007
% bleeding	1.286	3.406	1.286	8.177
Periodontal probing depth (PPD)	0.464	1.667	0.493	3.101
Loss of attachment	−0.536	0.001	−0.521	1.372

**Table 3:** Evolution of the periodontal parameters with *p* values after one year post-treatment.

Variable	SRP Mean	*p* Values SRP	LANAP Mean	*p* Values LANAP
% plaque	3.714	0.026	1.714	0.102
% bleeding	2.714	0.050 *	1.857	0.071
Periodontal probing depth (PPD)	0.543	0.006 *	0.521	0.165
Loss of attachment	−0.636	0.016	−0.564	0.054

(*) statistically significant.

**Table 4:** The clinical parameters before treatment and after 1 year post-treatment.

Variable	Mean Before Treatments		After 1 Year Post-Treatments			
	SRP Group	LANAP Group	SRP	*p* Values SRP	LANAP	*p* Values LANAP
% plaque	8.786	6.643	3.714	0.026	1.714	0.102
% bleeding	9.429	9.571	2.714	0.05 *	1.857	0.071
Periodontal probing depth (PPD)	0.629	0.693	0.543	0.006 *	0.521	0.165
Loss of attachment	−0.671	−0.721	−0.636	0.016	−0.564	0.054

(*) statistically significant.

## Data Availability

The original contributions presented in the study are included in the article, further inquiries can be directed to the corresponding author.

## Abbreviations

The following abbreviations are used in this manuscript:

SRP	Scaling and root planing
LANAP	Laser-assisted new attachment procedure
Nd:YAG laser	Neodymium-doped yttrium aluminum garnet laser
CAL PPD	Clinical attachment loss Periodontal probing depth
OPG	Orthopantomogram
